# Comprehensive immune profiling of SARS-CoV-2 infected kidney transplant patients

**DOI:** 10.3389/frtra.2023.1261023

**Published:** 2023-11-20

**Authors:** Franz Fenninger, Karen R. Sherwood, Vivian Wu, Paaksum Wong, Mari L. DeMarco, Meng Wang, Vincent Benedicto, Krishna A. Dwarka, Oliver P. Günther, Logan Tate, Eric Yoshida, Paul A. Keown, Matthew Kadatz, James H. Lan

**Affiliations:** ^1^ Department of Medicine, University of British Columbia, Vancouver, BC, Canada; ^2^ Department of Pathology and Laboratory Medicine, University of British Columbia, Vancouver, BC, Canada; ^3^ Department of Pathology and Laboratory Medicine, Providence Health Care, Vancouver, BC, Canada; ^4^ BC Provincial Immunology Laboratory, Vancouver Coastal Health, Vancouver, BC, Canada; ^5^ Günther Analytics, Vancouver, BC, Canada; ^6^ Division of Gastroenterology, University of British Columbia, Vancouver, BC, Canada; ^7^ Division of Nephrology, University of British Columbia, Vancouver, BC, Canada

**Keywords:** kidney transplantation, SARS-CoV-2, COVID-19, chronic kidney disease, immune profiling, immunophenotyping, TCR sequencing, cytokine profiling

## Abstract

**Introduction:**

The immune responses of kidney transplant recipients against SARS-CoV-2 remains under studied.

**Methods:**

In this prospective pilot study, we performed comprehensive immune profiling using cellular, proteomic, and serologic assays on a cohort of 9 kidney transplant recipients and 12 non-transplant individuals diagnosed with COVID-19.

**Results:**

Our data show that in addition to having reduced SARS-CoV-2 specific antibody levels, kidney transplant recipients exhibited significant cellular differences including a decrease in naïve—but increase in effector T cells, a high number of CD28+ CD4 effector memory T cells, and increased CD8 T memory stem cells compared with non-transplant patients. Furthermore, transplant patients had lower concentrations of serum cytokine MIP-1β as well as a less diverse T cell receptor repertoire.

**Conclusion:**

Overall, our results show that compared to non-transplant patients, kidney transplant recipients with SARS-CoV-2 infection exhibit an immunophenotype that is reminiscent of the immune signature observed in patients with severe COVID-19.

## 1. Introduction

The SARS-CoV-2 pandemic has a disproportionate impact on solid organ transplant (SOT) recipients. Compared to the general population, transplant patients are more susceptible to SARS-CoV-2 and more likely to experience severe outcomes (1–3). Patient characteristics such as age, comorbidities, and immunosuppressive regimen have been associated with an increased risk of severe disease (4). However, the mechanisms contributing to the increased risk in SOT patients remain unclear (5), as early reports indicate that patients most severely affected by SARS-CoV-2 exhibit an exaggerated inflammatory response which may paradoxically benefit from immunomodulation (6, 7).

Outcomes of SOT patients with SARS-CoV-2 relative to none-immunosuppressed individuals remain uncertain. Several studies comparing SOT recipients and non-transplant patients with SARS-CoV-2 infection suggest that adverse outcomes and mortality are increased in SOT patients (8, 9). However, when adjusting for comorbidities outcomes become more comparable (5) and some smaller studies did not find any differences between the two cohorts at all (10, 11).

Identification of distinct immune signatures of SOT patients against SARS-CoV-2 offers mechanistic insights into the dysregulated immunity of immunosuppressed individuals with COVID-19. One study reported that compared to uninfected transplant patients, SOT recipients with symptomatic COVID-19 had fewer lymphocytes including memory CD4 and CD8 T cells as well as a lower number of anergic and senescent CD8 T cells but a greater frequency of activated B cells (12). SOT recipients on immunosuppression (IS) are found to be capable of forming SARS-CoV-2-specific antibodies even in severe cases of disease (13). Furthermore, several groups found that inflammatory markers and IL-6 were similar in hospitalized patients of both cohorts (14, 15) and correlated with disease severity (16).

Despite the availability of COVID vaccines, a significant number of transplant patients remain unvaccinated (17). In this study, we set out to better characterize this immune response in an unvaccinated patient cohort through use of a multi-omics approach which encompasses quantification of serum antibody levels and immunoglobulin isotypes, cell subtype immunophenotying, serum cytokine profiling and T cell receptor (TCR) sequencing in unvaccinated kidney transplant recipients and non-immunosuppressed individuals diagnosed with symptomatic COVID-19.

## 2. Material and methods

### 2.1. Study design

This was a prospective cohort study with a primary objective to evaluate and compare the immunological characteristics between kidney transplant recipients and non-transplant patients admitted to the hospital with symptomatic COVID-19. The study was approved by the institution's institutional review board (#H20-01715).

Both kidney transplant and non-transplant patients received standard care treatment for COVID-19, comprising supplemental oxygen, high-flow nasal cannula support, mechanical ventilation, antibiotics, antiviral agents, immunomodulating medications, vasopressor support, and renal replacement therapy, as determined by the primary care team.

### 2.2. Patient population

The study was performed at Vancouver General Hospital in British Columbia, Canada. Patients were recruited at the time of a confirmed positive nucleic acid amplification (NAA) test, tested by the British Columbia Centre for Disease Control (BCCDC), between November 2020 and June 2021, and who were >18 years old. Samples were collected on day of recruitment, and scheduled for 7, 14, 28 and 90 days of follow-up, including both in-patient and out-patient sample collection. Due to logistical difficulties of sample collection during the pandemic, longitudinal sampling was heterogenous. In this study we therefore decided to only select the first time point for each patient to undergo testing and compared the assay results of kidney transplant recipients to nontransplant controls.

### 2.3. Sample/data collection

Whole blood was collected using BD Vacutainer™ Plastic Blood Collection Tubes with Sodium Heparin or EDTA (Fisher Scientific). Sera for immunoglobulin assays and cytokine immunoassay, PBMCs for flow cytometric analysis, and buffy coats for DNA isolation for TCR sequencing were isolated and frozen. Genomic DNA was isolated from frozen buffy coat using the QIAsymphony DNA Mini Kit (Qiagen). Peripheral blood mononuclear cells (PBMC) were isolated using Lymphoprep (STEMCELL Technologies) and stored in liquid nitrogen for later flow cytometric analysis.

### 2.4. COVID plus antibody assay (IgG)

Sera was run using the LabScreen TM COVID PLUS antibody detection assay (ThermoFisher). Target proteins include: Full Spike extracellular domain; Spike S1; Spike Receptor Binding Domain (RBD); Spike S2; and Nucleocapsid (NC) protein. Additionally, the kit incorporates Spike S1 fragments from six other coronaviruses, (CoV-229E, HCoV-HKU1, HCoVNL63, HCoV-OC43, MERS-CoV and SARS-CoV). Antibody detection on antigen coated microparticles was performed per manufacturer's protocol. Specimens were analyzed on a Luminex LABScan® 100 instrument (Luminex Corp. Austin, Tx.). Final analysis of MFI (Mean Fluorescence Intensity) was performed using Microsoft Excel.

### 2.5. ImmunIQ assay (IgG, IgM, IgA)

Sera was also run on a quantitative mass spectrometry assay that measures anti-SARS-CoV-2 IgG, IgA and IgM isotypes. Four microliters of serum are diluted in buffer and added to RBD-coated magnetic beads; following incubation and washing, bound antibodies are eluted for reduction, alkylation and proteolytic digestion with trypsin. Analysis is performed by high performance liquid chromatography mass spectrometry (Shimadzu LC 20AD LC system coupled to a SCIEX 5,500 triple quadrupole mass spectrometer), where quantotypic and isotype-specific human IgG, IgM and IgA sequences are detected, and quantified via the inclusion of isotopically labeled internal standard peptides for each isotype. The concentration of the internal standards was assigned against the ERM-DA470K certified reference material, following our previously published approach (18). Data was analyzed using Analyst (SCIEX v.1.6) and Skyline (v.20.2).

### 2.6. Flow immune profiling cytometry (immunophenotyping)

PBMCs were thawed and left overnight in RPMI media to recover and then stained with Fixable Viability Stain 620 (BD Horizon™), and split for staining with three different panels. These include: (1) the Dri Treg Panel (BD Horizon™), (2) the Dri Memory T-Cell Panel (BD Horizon™), supplemented with drop-in antibodies CD28, HLA DR, CD195, CD279 and CD38 (all BD Pharmingen™), and (3) a custom B cell panel consisting of the antibodies IgM, CD3, CD24, CD19, HLA-DR, CD10, IgD, CD38, CD138, CD27, IgG and CD20 (all BD Pharmingen™). For the Dri Treg Panel the cells were fixed and permeabilized using Cytofix/Cytoperm (BD). Data was acquired on a CytoFLEX Flow Cytometer (Beckman Coulter) and analysed using Kaluza (Beckman Coulter) and FlowJo (BD) and visualized using the ggplot2 package of RStudio.

### 2.7. ProcartaPlex human cytokine immunoassay

Patient sera was run using the Invitrogen™ ProcartaPlex™ Human Cytokine Storm 21-plex Immunoassay (ThermoFisher). Target cytokines included G-CSF (CSF-3), GM-CSF, IFN alpha, IFN gamma, IL-1 beta, IL-2, IL-4, IL-5, IL-6, IL-8 (CXCL8), IL-10, IL-12p70, IL-13, IL-17A (CTLA-8), IL-18, IP-10 (CXCL10), MCP-1 (CCL2), MIP-1 α (CCL3), MIP-1 β (CCL4), TNF alpha, TNF beta. Cytokine detection on magnetic microparticles was performed as per manufacturer protocol and suggestions of the Field Application Specialist. Briefly, the kit was allowed to warm to room temperature. Supernatant samples were then thawed and clarified by centrifugation at 10,000 g for 10 min. 3 vials of lyophilized standards were each reconstituted with 50 ul of 1× Universal Assay Buffer, then pooled and topped up with 100 ul of Universal Assay Buffer to make a working 250 ul standard containing all 21 proteins. A 1:4 serial dilution was performed for a 7-pt standard curve with varying S1–S7 concentrations for each target and a background tube. Specimens were analyzed on a Luminex LABScan® 100 instrument. Data analysis was performed using ThermoFisher Lab App and Excel 2013.

### 2.8. TCRβ sequencing

CDR3 region of rearranged TCRβ genes were sequenced using the immunoSEQ hsTCRB assay (Adaptive Biotechnologies, WA) following manufacturer protocol. Briefly, PCR amplification of the CDR3 region was performed using multiplex PCR with V- and J-gene primers and universal adaptor sequences. Libraries were sequenced on an Illumina MiSeq System (Illumina, CA) and output files were transferred to Adaptive Biotechnologies for TCRβ CDR3 sequence analysis using the immunoSEQ® 3.0 Analyzer (Adaptive Biotechnologies). A rearrangement was defined as a particular nucleotide sequence considering CDR3 + V region + J region. A template corresponded to a copy of a specific rearrangement. Productive clonality was calculated for a sample as 1 minus normalized Shannon's entropy for all productive (in-frame) rearrangements using the immunoSEQ® 3.0 Analyzer (Adaptive Biotechnologies). SARS-CoV-2 associated TCRβ rearrangements were found in the ImmuneCODE database using the immunoSEQ T-MAP COVID search tool (19) to determine the *bio-identity hits* defined as the number of unique rearrangements in the sample with a functional (CDR3 amino acid + V Gene + J Gene) match in the reference library; *rearrangement hits* defined as the number of unique rearrangements in the sample with a base-by-base match in the reference library, and *total hits* defined as the total number of unique rearrangements in the sample with either a rearrangement or bio-identity match in the reference library (19).

### 2.9. Data analysis

Statistical significance between the Non-Tx and the KTx group for demographic and clinical variables were determined using a Mann–Whitney *U*-test for numerical and a chi-squared test for categorical variables. The statistical significance of the assay results was calculated using a linear regression model in R (R Foundation, Vienna). A result was considered statistically significant if the *p*-value was ≤0.05. To determine the importance of immune features between transplant and non-transplant patients, a random forest analysis was performed using the function RandomForest() from the R package randomForest in classification mode and the mean gini decrease and mean accuracy decrease were calculated. Mtry = 55 was evaluated with the train() function from the caret package and for imputation of the missing data the rfImpute() function was used.

## 3. Results

**Figure 1:**
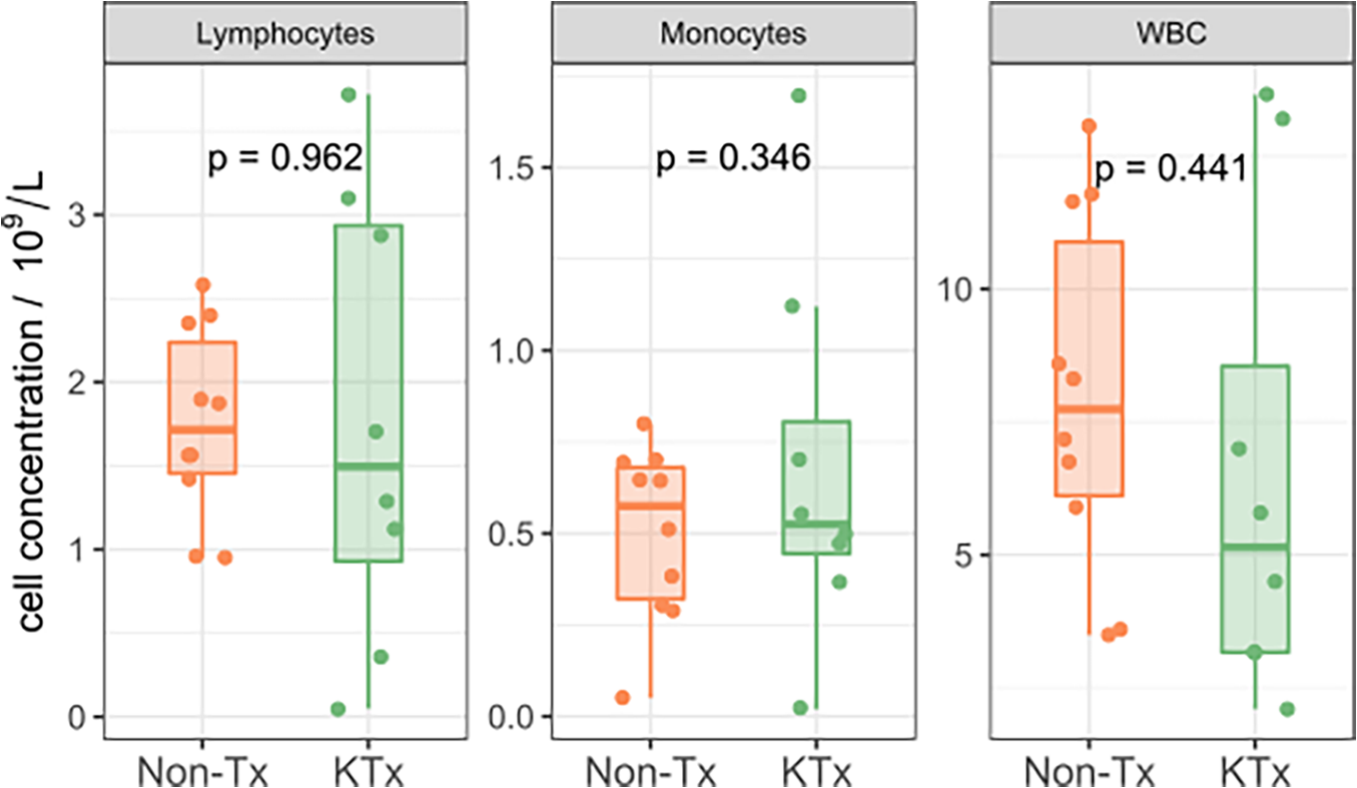
CBC with differential quantifying lymphocytes, monocytes and white blood cells.

Five immune monitoring assays were combined to comprehensively characterize the immune phenotype of 21 unvaccinated patients diagnosed with SARS-CoV-2. 12 patients were non-transplant patients not on immunosuppressive medications (Non-Tx). The study included 9 kidney transplant recipients maintained on immunosuppression (KTx) with a median transplant time of approximately 4 years. 16.7% of the Non-Tx cohort were female with a median age of 54.0 (Q1: 30.8, Q3: 57.5) years compared to 55.6% and a median age of 65.0 (Q1: 62.0, Q3: 68.0) years in the KTx cohort. The median number of days between the first SARS-CoV-2 positive NAA test and the sample taken for our analysis was 13.0 (Q1: 6.5, Q3: 17.0) in the Non-Tx group vs. 20.0 (Q1: 7.0, Q3: 46.0) in the KTx cohort (Table 1). Complete blood count variables such as white blood cell-, lymphocyte- and monocyte count did not show a significant difference between the two groups (Figure 1). Due to limited sample availability, some patients (*n* = 7) were not able to be run on all 5 assays (Supplementary Figure S1). While the immune phenotype between the two groups was vastly different, patients from both groups experienced mild to moderate disease and none of the patients progressed to require mechanic ventilation or experienced death after their immunophenotype testing.

**Table 1:** Demographics and clinical variables of study participants.

	Not transplanted (*N* = 12)	Transplanted (*N* = 9)	Total (*N* = 21)	*p* value
Sex				0.061
F	2 (16.7%)	5 (55.6%)	7 (33.3%)	
M	10 (83.3%)	4 (44.4%)	14 (66.7%)	
Blood type				0.986
Missing data	2	0	2	
A	3 (30.0%)	2 (22.2%)	5 (26.3%)	
AB	1 (10.0%)	1 (11.1%)	2 (10.5%)	
B	3 (30.0%)	3 (33.3%)	6 (31.6%)	
O	3 (30.0%)	3 (33.3%)	6 (31.6%)	
Age				0.026
Median	54.0	65.0	57.0	
Q1,Q3	30.8, 57.5	62.0, 68.0	44.0, 65.0	
Days post transplant				
Missing data	12	0	12	
Median	NA	1,452.0	NA	
Q1,Q3	NA	1,048.0, 1,793.0	NA	
Days post PCR+				0.226
Median	13.0	20.0	15.0	
Q1,Q3	6.5, 17.0	7.0, 46.0	7.0, 21.0	
WBC				0.286
Missing data	2	1	3	
Median	7.7	5.1	6.9	
Q1,Q3	6.1, 10.9	3.2, 8.6	3.8, 10.9	
Lymphocytes				0.929
Missing data	2	1	3	
Median	1.7	1.5	1.6	
Q1,Q3	1.5, 2.2	0.9, 2.9	1.2, 2.4	
Monocytes				0.689
Missing data	2	1	3	
Median	0.6	0.5	0.5	
Q1,Q3	0.3, 0.7	0.4, 0.8	0.4, 0.7	

### 3.1. SARS-CoV-2 serology, antibody quantification and immunoglobulin subclass dynamics

**Figure 2:**
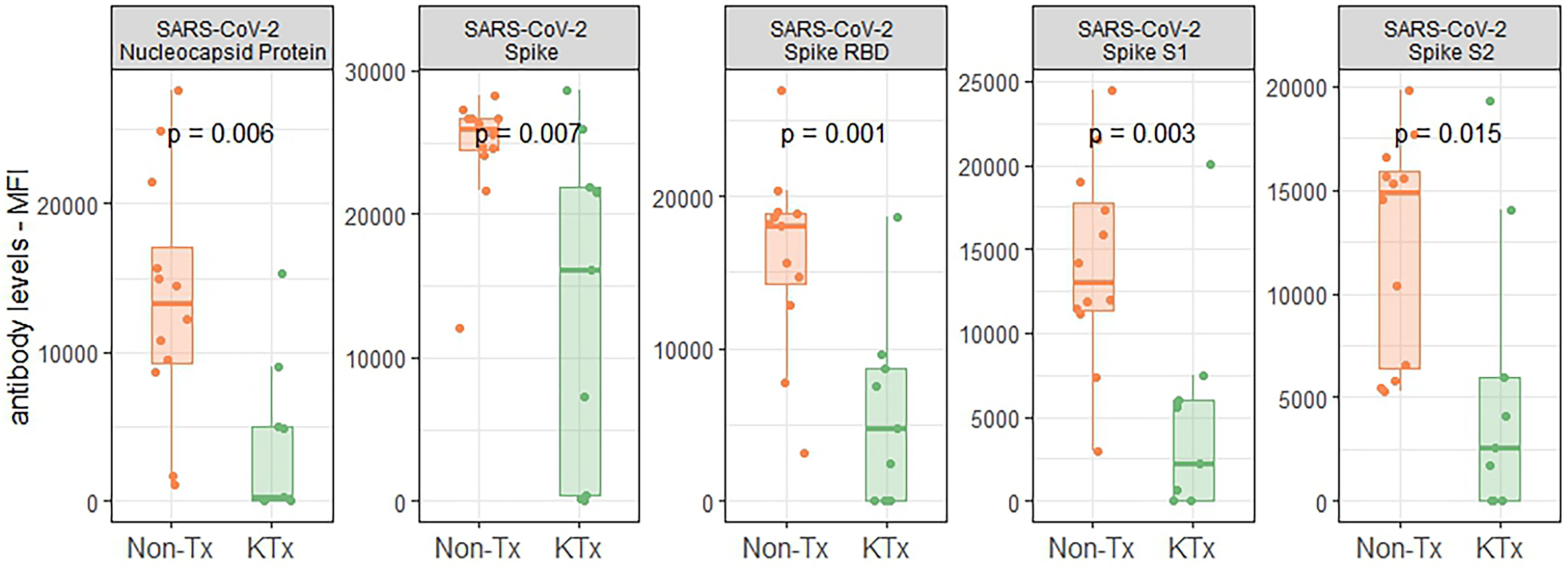
Reactivity of serum antibodies to SARS-CoV-2 proteins determined using a luminex based assay.

**Figure 3:**
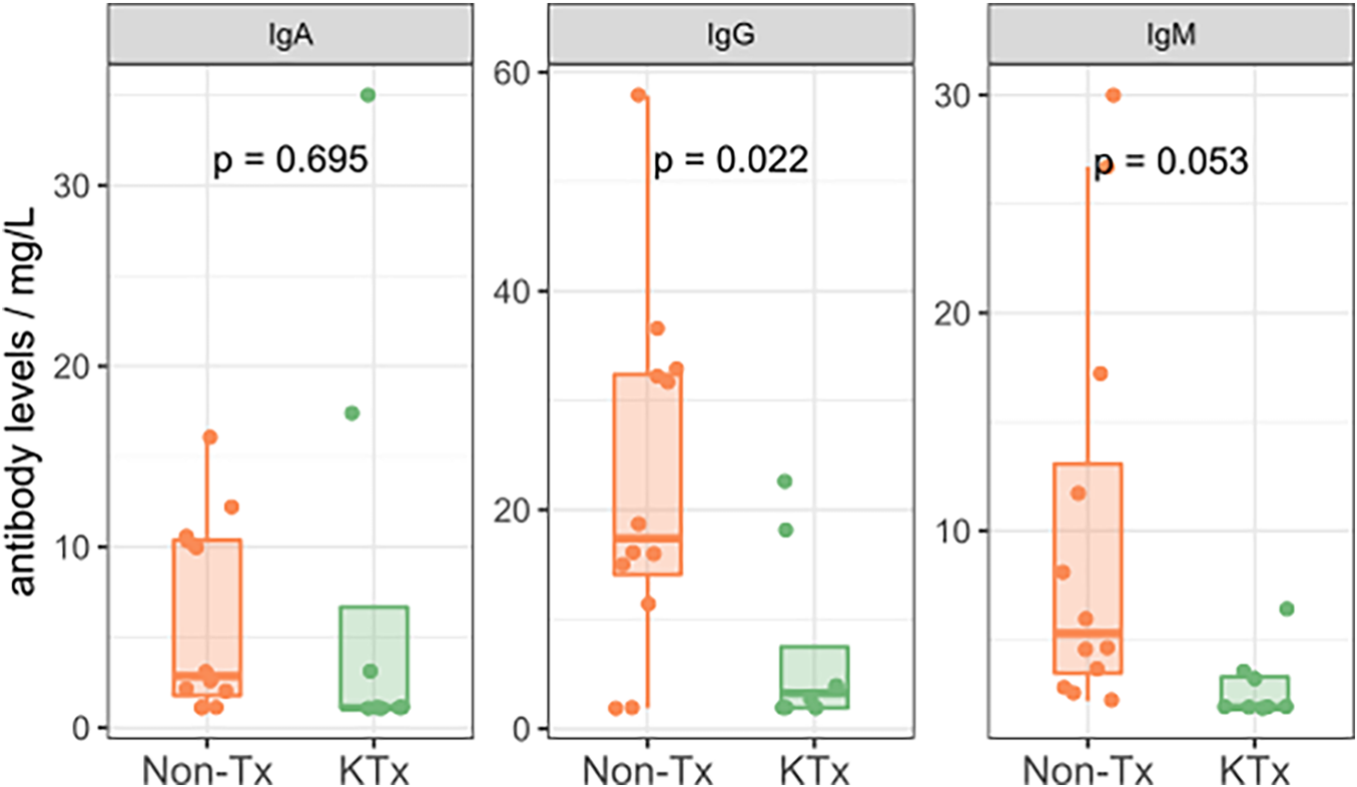
Isotypes levels of SARS-CoV-2 specific antibodies as determined by the immunIQ assay.

Semi-quantitative multiplexed analysis of IgG serum antibodies specific to SARS-CoV-2 proteins, other endemic and novel human coronaviruses were assessed. The KTx group produced significantly lower levels of SARS-CoV-2-specific antibodies (SARS-CoV-2-Nucleocapsid, SARS-CoV-2 Spike, SARS-CoV-2 Spike RBD, SARS-CoV-2 Spike S1 or SARS-CoV-2 Spike S2, Figure 2) while antibodies against other coronaviruses were similar in MFI levels between the two groups (Supplementary Figure S2). To further characterize the humoral response, we performed an in-house ImmunIQ assay which quantitatively detects anti-SARS-CoV-2 IgG, IgA, and IgM isotypes by using the RBD domain from the spike protein as the capture antigen. Using this assay, IgG antibody levels were significantly lower- and IgM antibodies mildly reduced in the KTx cohort compared to the Non-Tx group. In contrast, IgA antibodies showed no significant difference between the two groups (Figure 3).

### 3.2. Cellular immunophenotyping of key immune cell subsets

**Figure 4:**
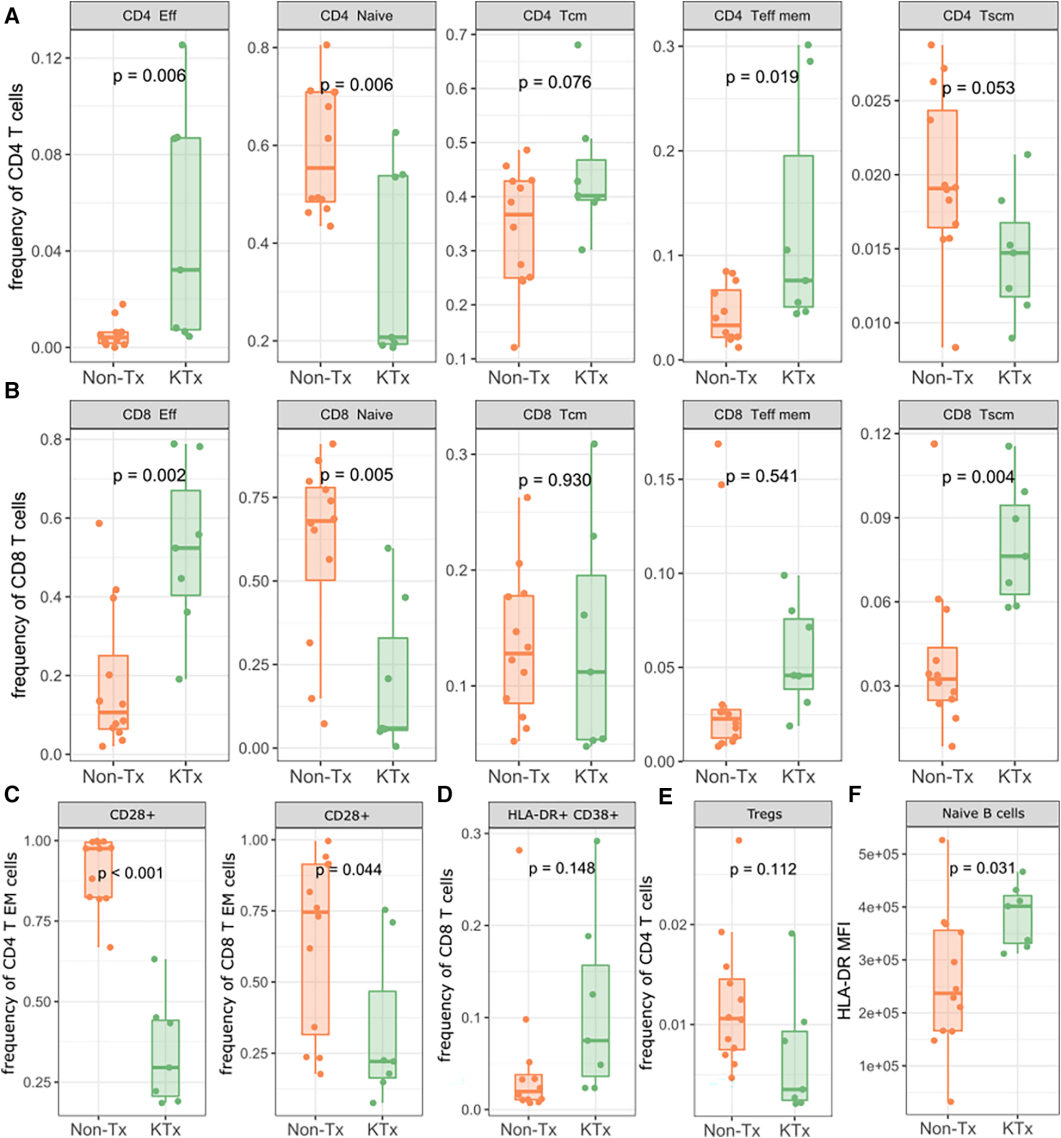
Lymphocyte subpopulation frequencies as determined by flow cytometry. (**A**) Subpopulation frequencies of CD4 T cells. (**B**) Subpopulation frequencies of CD8 T cells. (**C**) Frequencies of CD28+ CD4/CD8 effector memory T cells. (**D**) Frequencies of HLA-DR + CD38+ CD8+ T cells. (**E**) Frequencies of regulatory T cells. (**F**) MFI of HLA-DR on naïve B cells. Eff: Effector; Tcm: T central memory; Teff mem: T effector memory; Tscm: T memory stem; Treg: regulatory T.

Immune cell phenotyping from whole blood was performed using PBMCs with panels specific for T regulatory, T memory and B cells. The overall CD4 and CD8 T cell as well as B cell frequencies were not significantly different between the KTx and Non-Tx cohort (Supplementary Figure S3). However, CD4 effector and effector memory T cells were significantly increased in the KTx patients while CD4 naïve T cell frequencies were reduced (Figure 4A). We observed a similar pattern in the CD8 T cell subset in which effector T cell frequencies were also significantly increased while naïve T cell frequencies were reduced. In contrast to the CD4 T cells, in the CD8 T cell subset T memory stem (TSCM) cells were also increased in frequency in the KTx cohort (Figure 4B). Patients in the KTx cohort showed loss of CD28 expression as observed by the strong reduction of CD28+ T cells, particularly in the effector memory subset of both CD4 and CD8 T cells (Figure 4C). Furthermore, frequencies HLA-DR + CD38 + cells within the CD8 T cell population were mildly increased in the KTx cohort (Figure 4D) while Tregs showed a decreasing trend (Figure 4E). Lastly, while B cell population were comparable between the two groups (Supplementary Figure S4), HLA-DR expression levels were significantly upregulated in, among others, naïve B cells in the KTx cohort (Figure 4F).

### 3.3. Serum cytokines profiling

**Figure 5:**
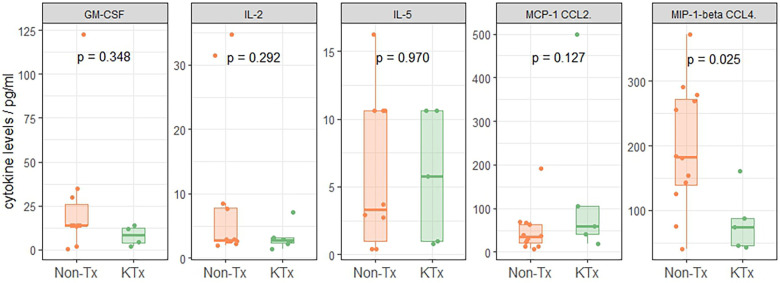
Serum-cytokine levels using the proCartaPlex luminex panel.

21 cytokines commonly associated with the previously described COVID-19 ‘cytokine storm’ phenomenon (20–23) were evaluated using the ProCartaPlex Luminex panel. Serum cytokines of the two patient groups were similar except for MIP-1β (CCL4) levels which were reduced in sera of KTx patients (Figure 5, Supplementary Figure S5), while Non-Tx patients had levels similar to reference values reported from healthy subjects (24).

### 3.4. T-Cell receptor sequencing for T-cell repertoire

**Figure 6:**
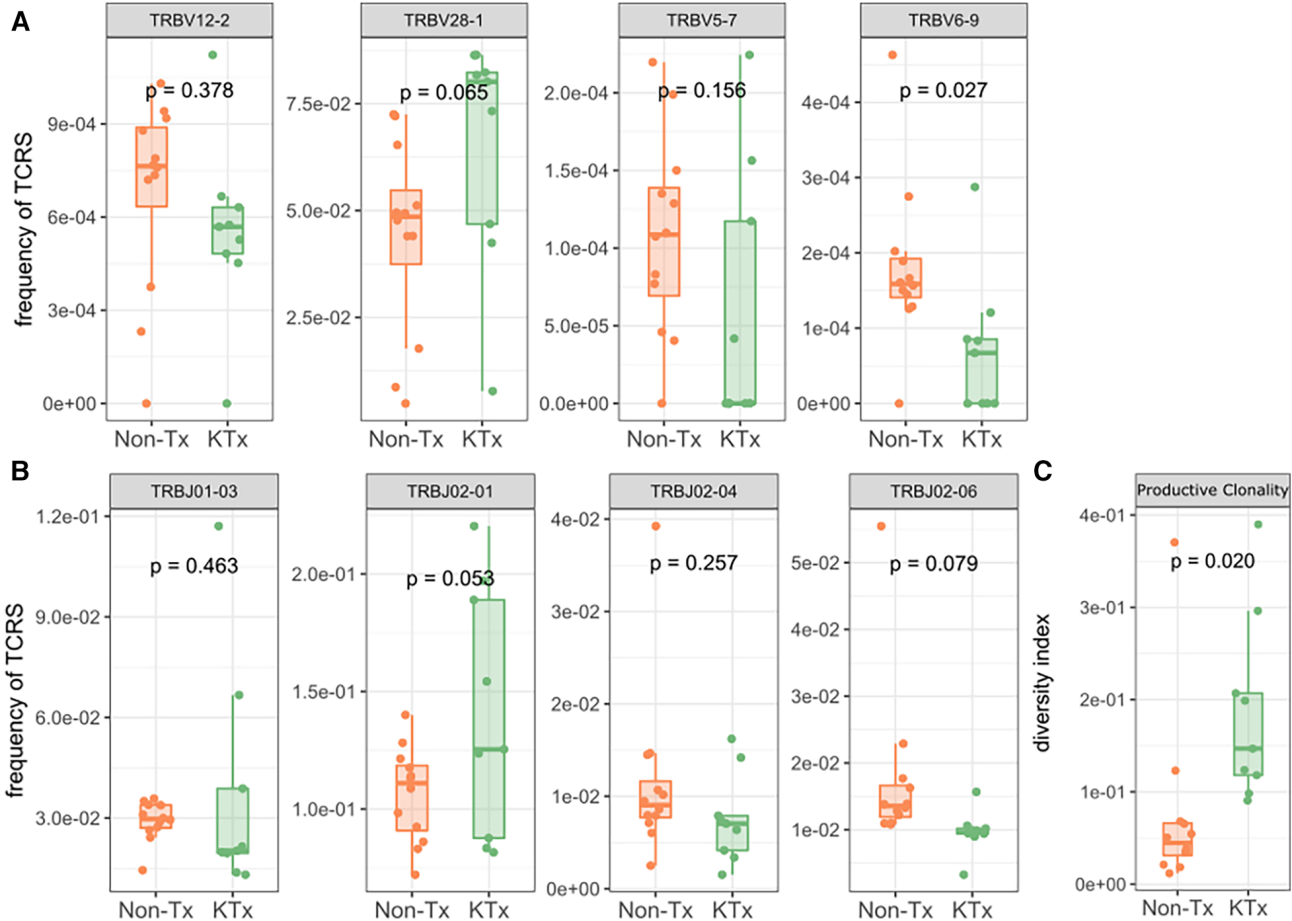
TRBV gene usage and productive clonality as determined by immunosequencing. (**A**) Usage of TRBV gene families. (**B**) Usage of TRBJ gene families. (**C**) Productive clonality of the TCR repertoire.

**Figure 7:**
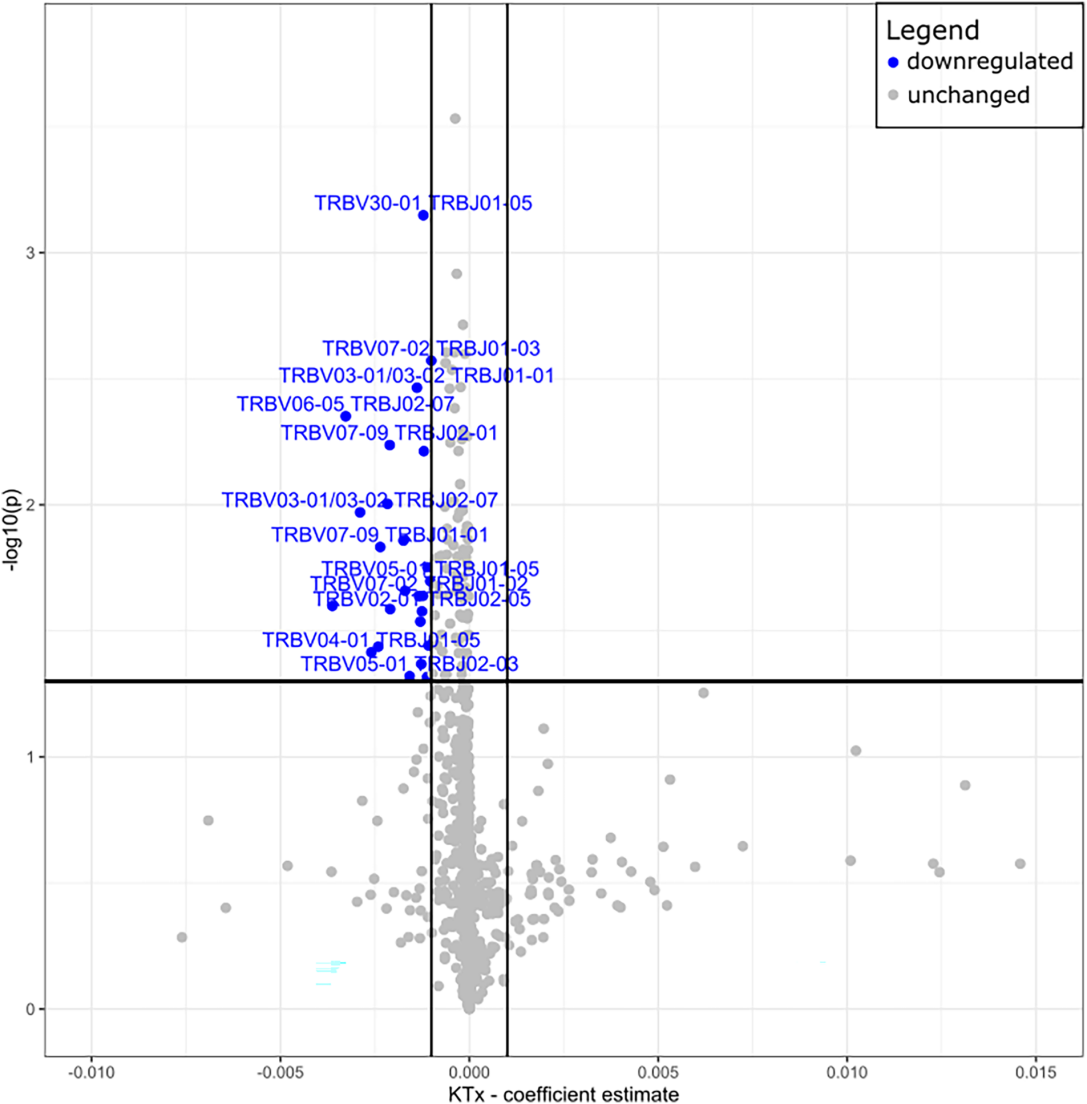
Volcano plot of TRBV—and TRBJ gene combination frequencies coefficient estimates of transplanted vs. non-transplanted patients. Blue = significantly decreased in KTx.

Peripheral T cell repertoire profiling was performed for human TCRβ on the two cohorts. No significant differences in the number of rearrangements or total template counts were identified (Supplementary Figure S6). We explored the usage of V and J gene segments and found a decrease in usage for most segments, except for TRBV28-1 and TRBJ02-01 amongst the KTx group. (Figures 6A,B). The KTx cohort showed a higher productive clonality, signifying a repertoire dominated by fewer rearrangements compared to a more polyclonal repertoire in patients without a transplant (Figure 6C). When examining combined VJ gene usage we did not find any combinations that were significantly upregulated, while several were decreased in the KTx cohort (blue dots in Figure 7). We also compared the number of SARS-CoV-2-specific TCRs using the immunoSEQ T-MAP COVID database (19) and did not find a significant difference in the bio-identity, rearrangement, and total hits between the two cohorts (Supplementary Figure S7).

### 3.5. Random forest

**Figure 8:**
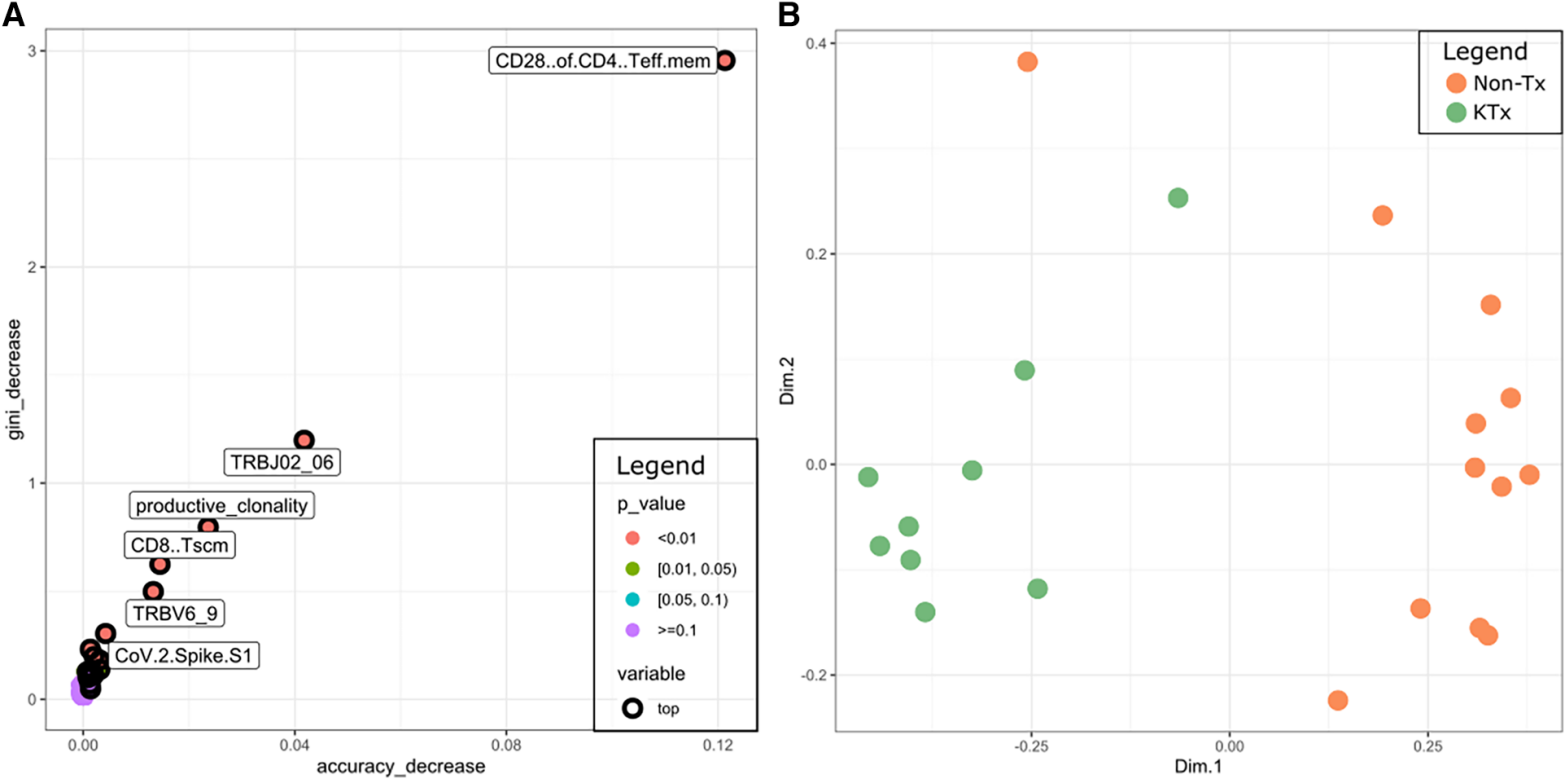
(**A**) Multi-way importance plot depicting the most important features of the random forest classifier. (**B**) MDS plot using the features of the random forest classifier.

Finally, we used the data from all five different assays to train a random forest classifier. The most prominent features were CD28 + of CD4 T EM cells (immunophenotyping), TRBJ2-6 (TCR sequencing), TRBV6-9 (TCR sequencing), productive clonality (TCR sequencing), CD8 TSCM cells (immunophenotyping), SARS-CoV-2 Spike S1 (antibody) (Figure 8A). A Multidimensional Scaling plot showed clear separation of the two cohorts suggesting it could potentially also be used as a classifier (Figure 8B).

## 4. Discussion

Solid organ transplant recipients represented a particularly vulnerable group during the SARS-CoV-2 pandemic, with multiple epidemiologic studies showing higher incidence rates of infection (25, 26) and increased transmission rates of the virus (27, 28) within this population. However, there is still controversy as to whether clinical outcomes differ between transplanted patients and the general population after adjusting for baseline comorbidities (5, 10, 11). We previously published a case report of a kidney transplant recipient whose SARS-CoV-2 infection in the first week post-transplantation did not significantly impair the patient's ability to mount a protective immune response (18). Here, we present, using a multi-omics approach, insights into the immune response of immunosuppressed renal transplant patients to COVID-19 infection.

Serological investigation using semi-quantitative IgG SARS-CoV-2 specific antibody targets revealed significantly lower SARS-CoV-2 spike protein specific IgG concentrations in the KTx cohort. Although median days post-positive test for the KTx cohort was 35 days vs. 12 days for the non-transplanted cohort in our study, a previously published IgG kinetics suggests that SARS-CoV-2 IgG levels increase in the immediate post-infection period, peaking approximately 3–7 weeks post infection and persisting for∼8 weeks (29). Thus, we would predict even higher IgG levels for the non-transplanted cohort if they had been measured at a median time period of 35 days post-transplant, which would further magnify differences in the anti-SARS-CoV-2 IgG responses between the two cohorts. The diminished humoral response in KTx patients might be related to the preferential suppression of IgM/IgG production from B cells/plasma cells by mycophenolate mofetil (MMF) (30, 31) and tacrolimus (32). Isotype-specific immunoglobulin tested also showed diminished isotype-specific SARS-CoV-2 antibody levels, particularly IgG antibodies in KTx patients, confirming observations from the semi-quantitative Luminex assay. While SARS-CoV-2 specific IgM antibodies were also lower in the KTx cohort, this was not statistically significant. IgA levels were comparable between cohorts, likely due to IgA being a mucous membrane secreted immunoglobulin and peripheral blood samples were used for this study to monitor systemically secreted immunoglobulins. Both cohorts had been exposed to endemic coronaviruses, as demonstrated by similar, low-level detection of non-SARS antibodies in both groups. Cytokine levels of all tested cytokine targets were similar between the two cohorts except for MIP-1β (CCL4), which was reduced in the KTx cohort. Previous studies have showed that SARS-CoV-2-reactive CD8^+^ T cells have a reduced capacity to secrete effector cytokines (specifically CCL4) (33) and reduced plasma levels of MIP-1β (CCL4) have been correlated with poor SARS-CoV-2 patient survival (34). Increased MCP-1 (CCL2) levels which have also been associated with unfavourable outcomes (35) were observed in our KTx cohort although the difference between the two groups was not statistically significant.

Compared with Non-Tx individuals, transplant recipients showed a general increase in effector T cell subsets and a decrease in naïve T cell subsets, while effector memory T cells were only increased in the CD4 subset. T memory stem (TSCM) cells were reduced in KTx patients in the CD4 T subsets but were present at a significantly higher frequency in the CD8 T cell subset. TSCM cells have the ability to self-renew and can reconstitute the full diversity of effector and memory T cell subsets (36). In particular, CD8 TSCM are detected early after antigenic challenge (i.e., vaccine) when effector T cells dominate the immune response (37). In our KTx cohort we observed the simultaneous increase of effector T cells with CD8 TSCM cells. Interestingly, in a biological setting of impaired humoral response (hematological malignancies), CD8 T cells have been shown to compensate for impaired humoral immunity in Covid-19 patients (38), presenting a possible mechanism by which the KTx patients have an altered B cell response while still retaining the higher frequency CD8 effector and CD8 TSCM subsets.

KTx patients also demonstrated an almost complete loss of CD28+ T cells, particularly in CD4 EM T cells. CD28 is a co-stimulatory molecule that binds to a B7 molecule on antigen-presenting-cells, along with the TCR interacting with a cognate antigen presented by the MHC complex. Repeated stimulation due to chronic clinical conditions lead to the downregulation of CD28 and eventually renders a cell anergic (39). End-stage renal disease (ESRD) and solid organ transplants have both been associated with an increase of CD28- T cells (and therefore a decrease of CD28+ T cells) (40) which could account for the loss of CD28+ T cells in our data. Furthermore, several pathologic conditions such as viral infections are also known to cause CD28 downregulation. A synergistic effect on CD28 downregulation between a history of ESRD/immunosuppression and SARS-CoV-2 infection is possible but we could not directly interrogate this effect in this study as this would require an uninfected control group. Nevertheless, the accumulation of CD28- senescent T cells has been associated with higher morbidity and mortality in COVID-19 patients (41).

The immunophenotyping data showed that KTx patients expressed more HLA-DR on B cells, particularly naïve B cells. This suggests that despite producing less SARS-CoV-2 specific antibodies, naïve B cells of KTx patient appear to be capable of presenting more antigen. Despite classically known as inefficient antigen presenters, B cells have shown to act as antigen presenting cells and activate CD4 and CD8 T cells (42) and could thereby contribute to the increased cellular immunity in the KTx cohort.

The TCR repertoire of the KTx patient showed a decreased usage of most V and J segments but did not correspond with any reports in the literature about VJ usage during COVID infection. The KTx patients also demonstrated an elevated TCR productive clonality (high abundance of restricted clones), but a less diverse TCR repertoire, a phenotype that fits with their highly differentiated T cells. Since this phenomenon has been documented previously in patients with chronic kidney disease (43) the increased clonality has most likely developed in the KTx patients during their CKD. The number of SARS-CoV-2 specific TCRs did not differ significantly between the two cohorts, suggesting that transplant patients maintained on immunosuppression were able to mount a COVID-specific T cell response comparable to that generated by non-immunosuppressed individuals. Given the lack of HLA typing data in the Non-Tx cohort, our TCR repertoire analysis was not able to account for the impact of HLA restriction to SARS-CoV-2 as recently performed by another group (44).

The random forest analysis demonstrates that the measured features can potentially be used to distinguish the two cohorts and that the key features for the classification were derived from the immunophenotyping and the TCR sequencing data. These initial results warrant additional validation in a prospective larger cohort, to confirm the random forest classifier.

Overall, our results show that compared to Non-Tx individuals, kidney transplant recipients with a SARS-CoV-2 infection mounted a limited humoral immune response, which appeared to be compensated with a robust cellular response. They also appeared to exhibit an immunophenotype that is reminiscent of more severe COVID-19 patients, reflected in the decreased naïve—but increased effector T cells, the accumulation of CD28- senescent T cells, and a cytokine profile of increased MCP-1(CCL2)/ reduced concentration of MIP-1β (CCL4), even though characterization by their symptoms using the WHO classification suggested otherwise. One limitation of our study is that it only reflects the profiles of surviving non-ICU patients. Even though no patients in our cohort died after enrollment, our study did not recruit patients presenting with severe disease requiring ICU support from the outset, which might have introduced a survival bias.

Further confirmation of these findings in a larger, prospective cohort as well as in other organ transplant cohorts, could better define the use and clinical utility of immune monitoring in guiding clinical management in immunosuppressed individuals with COVID-19 (45).

## Data Availability

The TCR sequencing data of this study are publicly available and can be found here: https://doi.org/10.21417/FF2023FT.

## Supplementary material

Supplementary Figure S1 Assays used for the different patients.

Supplementary Figure S2 Reactivity of serum antibodies to other (non-SARS-CoV2) human coronavirus proteins determined using a Luminex based assay.

Supplementary Figure S3 Lymphocyte population frequencies as determined by flow cytometry. (**A**) Frequencies of T cells. (**B**) Frequencies of CD4 and CD8 T cells. (**C**) Frequencies of B cells.

Supplementary Figure S4 B cell subpopulation frequencies as determined by flow cytometry. ASCs: Antibody-secreting cells.

Supplementary Figure S5 Heatmap showing the difference in serum cytokine levels measured using the ProCartaPlex Luminex panel in the KTx cohort compared to the Non-Tx cohort as a reference. * *p* < =0.05.

Supplementary Figure S6 Number of unique rearrangements and total templates as determined by TCR sequencing.

Supplementary Figure S7 Number of SARS-CoV-2 specific TCR bio-identity-, rearrangement- and total hits in the immunoSEQ T-MAP COVID database.
