# Supplementary figures and images for: Comprehensive immune profiling of SARS-CoV-2 infected kidney transplant patients
Source: Front Transplant. 2023 Nov 20;2:1261023. doi: 10.3389/frtra.2023.1261023 (PMC11235348; doi:10.3389/frtra.2023.1261023)

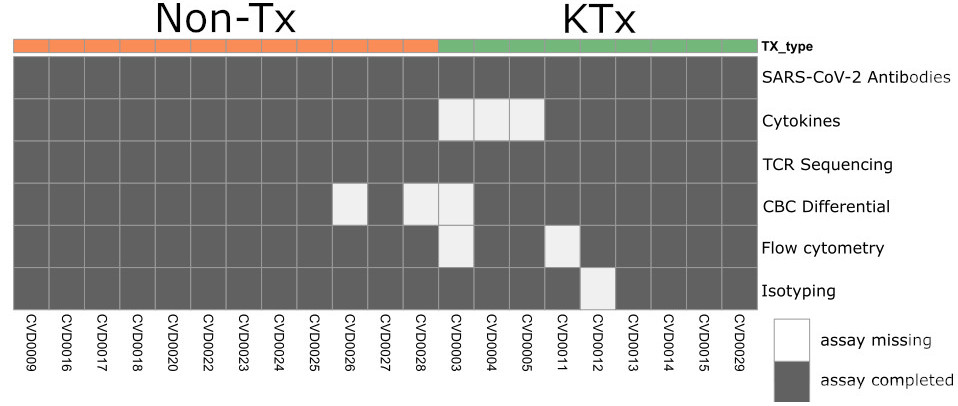

Supplement: Supplementary Figure S1 — Assays used for the different patients. [file Image1.jpg]

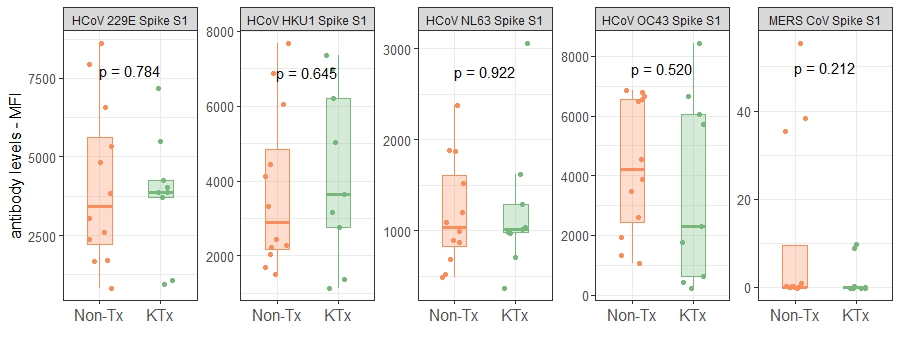

Supplement: Supplementary Figure S2 — Reactivity of serum antibodies to other (non-SARS-CoV2) human coronavirus proteins determined using a Luminex based assay. [file Image2.jpeg]

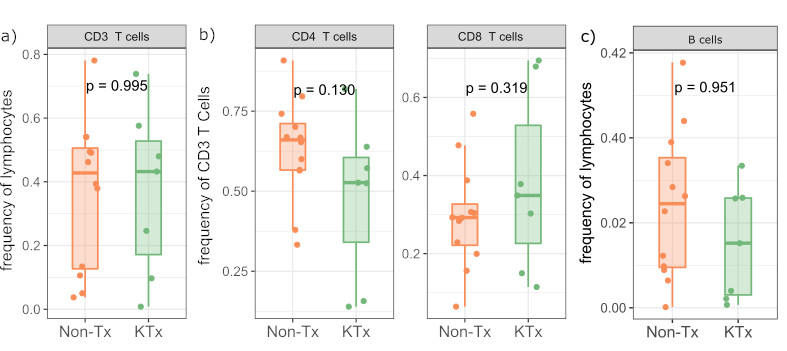

Supplement: Supplementary Figure S3 — Lymphocyte population frequencies as determined by flow cytometry. (A) Frequencies of T cells. (B) Frequencies of CD4 and CD8 T cells. (C) Frequencies of B cells. [file Image3.jpeg]

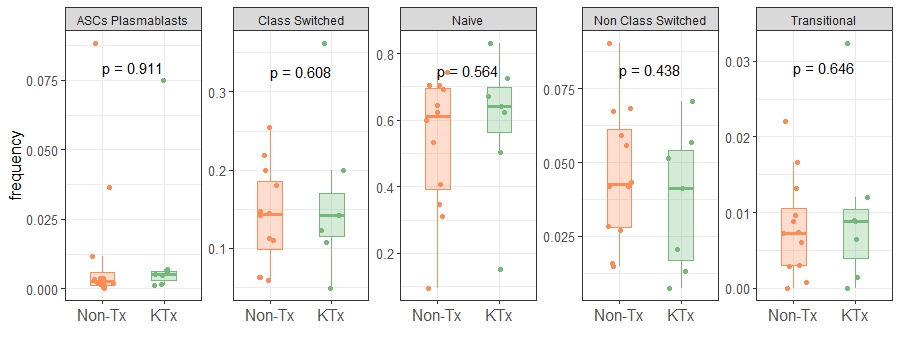

Supplement: Supplementary Figure S4 — B cell subpopulation frequencies as determined by flow cytometry. ASCs: Antibody-secreting cells. [file Image4.jpeg]

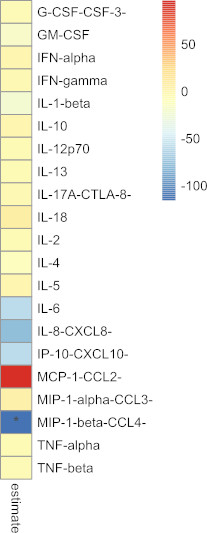

Supplement: Supplementary Figure S5 — Heatmap showing the difference in serum cytokine levels measured using the ProCartaPlex Luminex panel in the KTx cohort compared to the Non-Tx cohort as a reference. * p < =0.05. [file Image5.jpeg]

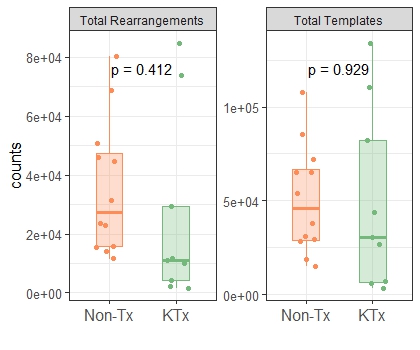

Supplement: Supplementary Figure S6 — Number of unique rearrangements and total templates as determined by TCR sequencing. [file Image6.jpeg]

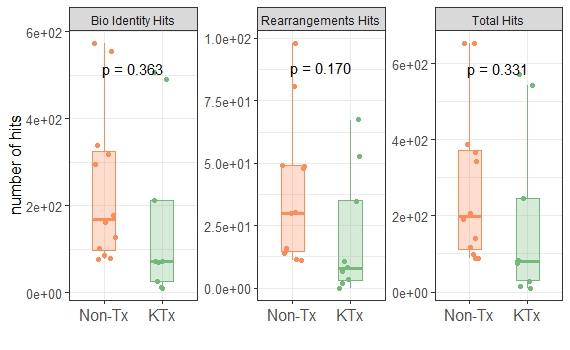

Supplement: Supplementary Figure S7 — Number of SARS-CoV-2 specific TCR bio-identity-, rearrangement- and total hits in the immunoSEQ T-MAP COVID database. [file Image7.jpeg]
